# Relationship between form-deprivation myopia and amblyopic deficit

**DOI:** 10.1016/j.visres.2026.108844

**Published:** 2026-04-30

**Authors:** Lisa A. Ostrin, Krista M. Beach, Nimesh B. Patel, Randolph J. Kwaw, Raman P. Sah, Vallabh E. Das

**Affiliations:** University of Houston College of Optometry, United States

**Keywords:** Amblyopia, Myopia, Animal models, Form-deprivation, Visual evoked potentials

## Abstract

This study investigated the relationship between form-deprivation myopia and amblyopic deficit. Five rhesus monkeys underwent monocular form-deprivation beginning at 24 ± 2 days of age. Lightweight helmets held a light-perception only Bangerter filter over the right eye and a plano lens over the left eye. Refractive error and axial length were measured biweekly using streak retinoscopy and noncontact biometry. After 135 ± 2 days of treatment, visual function in each eye was assessed under sedation by recording Visual Evoked Potentials (VEP) elicited by monocular stimulation with square wave gratings (spatial frequencies 0.25–16 cycles per degree). An Ocular Dominance Index (ODI), the difference in VEP amplitudes between form-deprived (amblyopic) eye stimulation and fellow (control) eye stimulation divided by the sum, quantified amblyopic deficits. At the end of treatment, mean spherical equivalent refraction and axial length were −0.43 ± 4.41 D and 16.66 ± 0.70 mm in form-deprived eyes and +1.75 ± 2.71 D and 16.07 ± 0.61 mm in fellow eyes, respectively. Anisometropia was highly variable, ranging from −6.13 to +2.75 D (mean −2.18 ± 3.60 D). All monkeys demonstrated a significant amblyopic deficit, with a mean ODI of −0.66 ± 0.11 (range: −0.43 to −0.81). Correlations between ODI and anisometropia and ODI and axial length were not significant in this small cohort. Monocular form-deprivation in young rhesus monkeys produced a robust amblyopic deficit as measured by VEPs, even in the absence of myopia. Findings indicate that form-deprivation induces central visual pathway deficits may not be correlated with the amplitude of form-deprivation myopia.

## 1. Introduction

Myopia is a complex refractive condition, most typically characterized by excessive axial elongation of the eye, leading to images being focused anterior to the retina. In humans, genetic and environmental factors contribute to myopia development, with altered visual experience during early life playing a particularly important role (Flitcroft et al., 2019). Among experimental paradigms used to study visually guided eye growth, form-deprivation has been one of the most robust and extensively characterized models (Smith et al., 1987, Troilo et al., 2019). In animal models, including chicks (Wallman, Turkel & Trachtman, 1978), guinea pigs (Howlett & McFadden, 2006), tree shrews (Norton, 1990), and rhesus monkeys (Smith et al., 1987, Smith & Hung, 2000), as well as in humans (Gusek-Schneider & Martus, 2001, Huo et al., 2012), degradation of retinal image quality during critical periods of development induces axial elongation and refractive error changes consistent with myopia (Raviola & Wiesel, 1985).

In addition to refractive change, form-deprivation during early development is associated with deficits in visual function (von Noorden, 1981). Reduced contrast sensitivity, decreased spatial resolution, and abnormal cortical responses have been documented following early monocular visual deprivation (Harwerth et al., 1981, Kiorpes, 2019, Wiesel & Hubel, 1963). In primates, these functional deficits often manifest as amblyopia, a neurodevelopmental disorder defined by reduced visual acuity or contrast sensitivity, without the presence of other ocular pathology (Levi, 2006). Numerous factors may contribute to amblyopia, including anisometropia, strabismus, or image degradation (Birch & Duffy, 2024, Hunter & Cotter, 2018). The severity of amblyopia does not necessarily scale with refractive error (McKee, Levi & Movshon, 2003).

Despite extensive use of form-deprivation paradigms to study the mechanisms of eye growth regulation in animal models, the relationship between induced refractive changes and the depth of amblyopia remains incompletely understood. While it is often assumed that greater degrees of form-deprivation myopia would lead to more pronounced amblyopic deficits, evidence supporting a direct correspondence between ocular elongation and cortical dysfunction is limited. Previous studies have suggested that form-deprivation myopia is largely driven by local retinal mechanisms (Smith et al., 2009), whereas amblyopia reflects central neural adaptations within the visual pathway (Kiorpes & Daw, 2018). Distinguishing ocular versus central effects is essential for a more complete understanding of how early visual experience shapes both ocular growth and visual function. Rhesus monkeys provide a particularly valuable model for addressing this question due to their close anatomical and functional similarity to the human visual system (Dubis et al., 2009, Qiao-Grider et al., 2007). Early postnatal visual development in rhesus monkeys parallels that of human infants, including sensitive periods for both emmetropization and cortical plasticity (Harwerth et al., 1990).

The purpose of the present study was to investigate whether the magnitude of form-deprivation myopia is associated with the severity of amblyopia in young rhesus monkeys. In human infants and in animals, objective electrophysiological measures, such as pattern visual evoked potentials (VEPs), offer a quantitative and reproducible method for assessing amblyopic deficits across a range of spatial frequencies, independent of behavioral performance (Arden et al., 1974). Therefore, by combining longitudinal measurements of refraction and ocular biometry with monocular pattern VEPs at the end of the deprivation period, this study aimed to test the hypothesis that greater anisometropia would correspond to a larger amblyopic deficit. Clarifying the relationship between ocular and cortical consequences of form-deprivation has important implications for understanding the mechanisms underlying visually guided eye growth and for interpreting functional outcomes in experimental models of myopia. Unlike prior studies that have examined refractive development and amblyopia largely in isolation, the present study directly relates experimentally manipulated eye growth with spatial frequency-resolved, VEP-defined amblyopic deficits in a primate model, providing a more integrated assessment of ocular and cortical consequences of early visual deprivation.

## 2. Methods

### 2.1. Animals

Infant rhesus monkeys (*Macaca mulatta*) were obtained at 2 weeks of age and reared in a non-human primate nursery (N = 5; 1 female:4 males). All rearing and experimental procedures were reviewed and approved by the University of Houston’s Institutional Animal Care and Use Committee and were in accordance with the ARVO Statement for the Use of Animals in Ophthalmic and Vision Research and the National Institutes of Health Guide for the Care and Use of Laboratory Animals.

Monkeys were reared under fluorescent lights (correlated color temperature = 3500 K; Philips TL735; Philips Lighting, Sommerset, NJ, USA) maintained on a 12-hour light/12-hour dark cycle (Smith & Hung, 1999). Average cage-level illuminance during the daily light cycle was 480 lx (range: 342–688 lx).

### 2.2. Form-deprivation

Monkeys underwent form-deprivation in the right eye starting at age 24 days, as described by Smith and Hung (Smith & Hung, 2000). In brief, monkeys were fit with a lightweight helmet that held a plano lens in front of both eyes. A Bangerter light-perception only occlusion foil diffuser (Fresnel Prism and Lens Co, Prairie, MN) was attached to the lens in front of the right eye, while the left eye served as the control. The light perception diffusers produce attenuation of contrast across spatial frequencies, with an effective cutoff below approximately 0.5–1 cyc/deg, thereby eliminating form vision while preserving only coarse luminance information (Smith & Hung, 2000). Monkeys wore the helmets at all times until 150 days of age, except during daily morning lens cleaning, in which the helmets were briefly removed, and during biweekly ocular measurements. The diffuser was removed for 10 days in one monkey (Monkey 4) during the early treatment period due to minor skin irritation. Following resolution, the diffuser was reapplied, and the animal completed the remainder of the protocol.

### 2.3. Ocular measurements

Refraction and biometry were measured every two weeks for the duration of the experiment. Details for these measurements have been outlined previously (Hung, Crawford & Smith, 1995, Smith & Hung, 1999). Briefly, monkeys were anesthetized with an intramuscular injection (30 mg/kg ketamine hydrochloride and 0.3 mg/kg acepromazine maleate) and cyclopleged with two drops of topical 1% tropicamide, separated by 10 min. Heart rate, body temperature, and partial pressure of oxygen were monitored throughout the measurement sessions.

Approximately 20 min after tropicamide instillation, spherocylindrical refraction was performed for each eye using streak retinoscopy (Welch Allyn Elite Streak Retinoscope; Welch Allyn Inc., Skaneateles Falls, NY) by two skilled examiners and averaged by matrix notation (Harris, 1988). The refraction of each eye was defined as the spherical-equivalent spectacle-plane refractive correction (Kee et al., 2004). Axial length was measured using an optical biometer (LenStar LS 900, Haag-Streit AG, Koeniz, Switzerland) in short-eye mode. At least five measurements were captured for each eye and averaged. The axial boundaries of the gates were manually adjusted in the optical A-scan to derive axial length, as described previously (She et al., 2022).

### 2.4. Visual evoked potentials

After 135 ± 2 days of treatment, Visual Evoked Potential (VEP) testing during monocular viewing (with the other eye occluded) was employed to test visual cortical responsivity through each eye (Hamilton et al., 2021, Norcia et al., 2015, Tychsen et al., 2004, Voyles & Kiorpes, 2016). VEP testing was performed while animals were sedated with a solution of ketamine (30 mg/kg) and xylazine (0.2–0.4 mg/kg). The diffusers were removed during VEP testing; thus, the spatial frequency content of the VEP stimuli (0.25–16 cyc/deg) was not optically limited at the time of measurement.

**Fig. 1.**
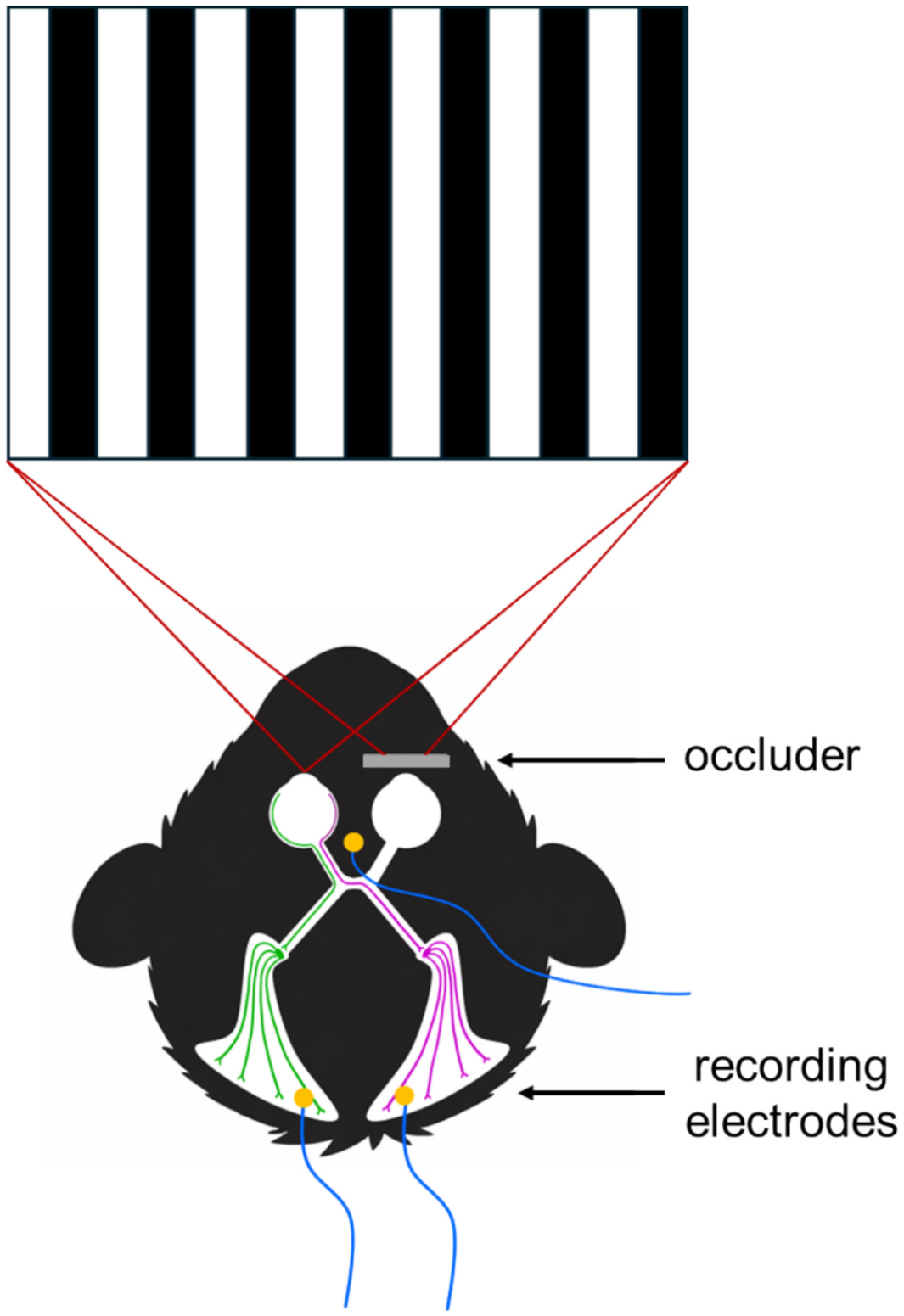
VEP set up.

Spherical equivalent refractive correction, adjusted for monitor viewing distance, was placed in front of each eye with a trial lens holder. The VEP set up is shown in Fig. 1. To record electroencephalography (EEG) signals, three reusable 10 mm gold cup electrodes were affixed to the scalp with conductive gel (neurodiagnostic electrode paste, Ten20) with one electrode placed on the forehead as a reference electrode and two electrodes placed a few millimeters above and lateral to the inion on either side to record responses from each V1 hemisphere. Signals were recorded simultaneously from both electrodes, and the responses were averaged for analysis.

Visual stimuli were generated using custom MATLAB software with the Psychophysics Toolbox extension and displayed on a 43 in. monitor (Dell Ultrasharp U4323QE) placed 82 cm from the monkey. The stimuli consisted of blocks of full contrast square wave gratings of varying spatial frequencies (0.25, 0.5, 1, 2, 4, 8, and 16 cyc/deg) that phase reversed (40 contrast reversals per spatial frequency per block at 6 reversals per second). Although different values of contrast reversal frequency were not tested systematically, the literature suggests that VEP spatial frequency limits are relatively unaffected by choice of stimulus frequency (Allen, Tyler & Norcia, 1996, Hamilton et al., 2021, Norcia et al., 2015). Further, our choice of 6 reversals/sec yielded strong signal well above the noise-floor at the different spatial frequencies tested. Data were also collected for a similar duration of a neutral gray presentation. The range of spatial frequencies used was informed from previous studies that described the development of grating acuity in monkeys, which is approximately 10–20 cyc/deg at ages corresponding to the current study and 30–50 cyc/deg in adults (Boothe, Williams, Kiorpes & Teller, 1980, Kiorpes, 1992). Spatial frequency blocks were presented in pseudo-random order, and each set of spatial frequencies was presented at least six times for each eye.

The EEG signals were recorded with Open-Ephys hardware and software, and data were analyzed offline using MATLAB custom software. The data analysis consisted of a Fourier transform of the raw EEG waveform for each spatial frequency and calculating the sum of the amplitude spectrum at the stimulus fundamental frequency and six additional harmonics (Fong et al., 2021). The choice of six additional harmonics was designed to capture as much of the VEP signal as possible (Norcia et al., 2015). The summed VEP amplitude from each presentation was averaged to obtain the final signal VEP estimate for that specific spatial frequency. In addition, baseline nonvisual activity (an estimate of noise) was calculated as the sum of the amplitude spectrum at frequencies 0.45 Hz away from the stimulus fundamental and the harmonics used for detecting the signal (Norcia et al., 2015). The values for baseline activity were routinely low and provided confirmation that measured stimulus driven responses were valid.

To quantify the amblyopic deficit, an Ocular Dominance Index (ODI) at each spatial frequency was calculated using the difference in VEP amplitudes recorded when monocularly stimulating the form-deprived (amblyopic) eye and the fellow (control) eye, divided by their sum (Eq. (1)). Mean ODI was calculated as the average across all spatial frequencies, as well as for low (0.25 to 0.5 cyc/deg), mid (1, 2, 4 cyc/deg), and high (8, 16 cyc/deg) spatial frequencies. Negative ODI values indicate reduced VEP amplitude in the form-deprived eye relative to the fellow eye, reflecting the presence of amblyopia. An ODI of 0 indicates equal responses from the two eyes; values approaching −1 indicate near-complete dominance of the fellow eye; values near +1 indicate the opposite.

(1) $$\text{Ocular}\;\text{dominance}\;\text{index}=\frac{{\text{VEP}}_{\text{form}\;\text{deprived}\;\text{eye}}-{\text{VEP}}_{\text{fellow}\;\text{eye}}}{{\text{VEP}}_{\text{form}\;\text{deprived}\;\text{eye}}+{\text{VEP}}_{\text{fellow}\;\text{eye}}}$$

### 2.5. Analysis

Data are presented as mean ± standard deviation unless otherwise noted. Statistical analyses were performed using IBM SPSS statistical software, version 21 (SPSS, Inc., Chicago, IL, USA). Refractions and axial lengths of the form-deprived eyes and fellow eyes over the treatment period were analyzed with repeated measures ANOVAs with two within-subjects factors (eye and time). Pearson correlation coefficient was used to assess the relationship between degree of anisometropia and axial length with cortical deficit.

## 3. Results

**Fig. 2.**
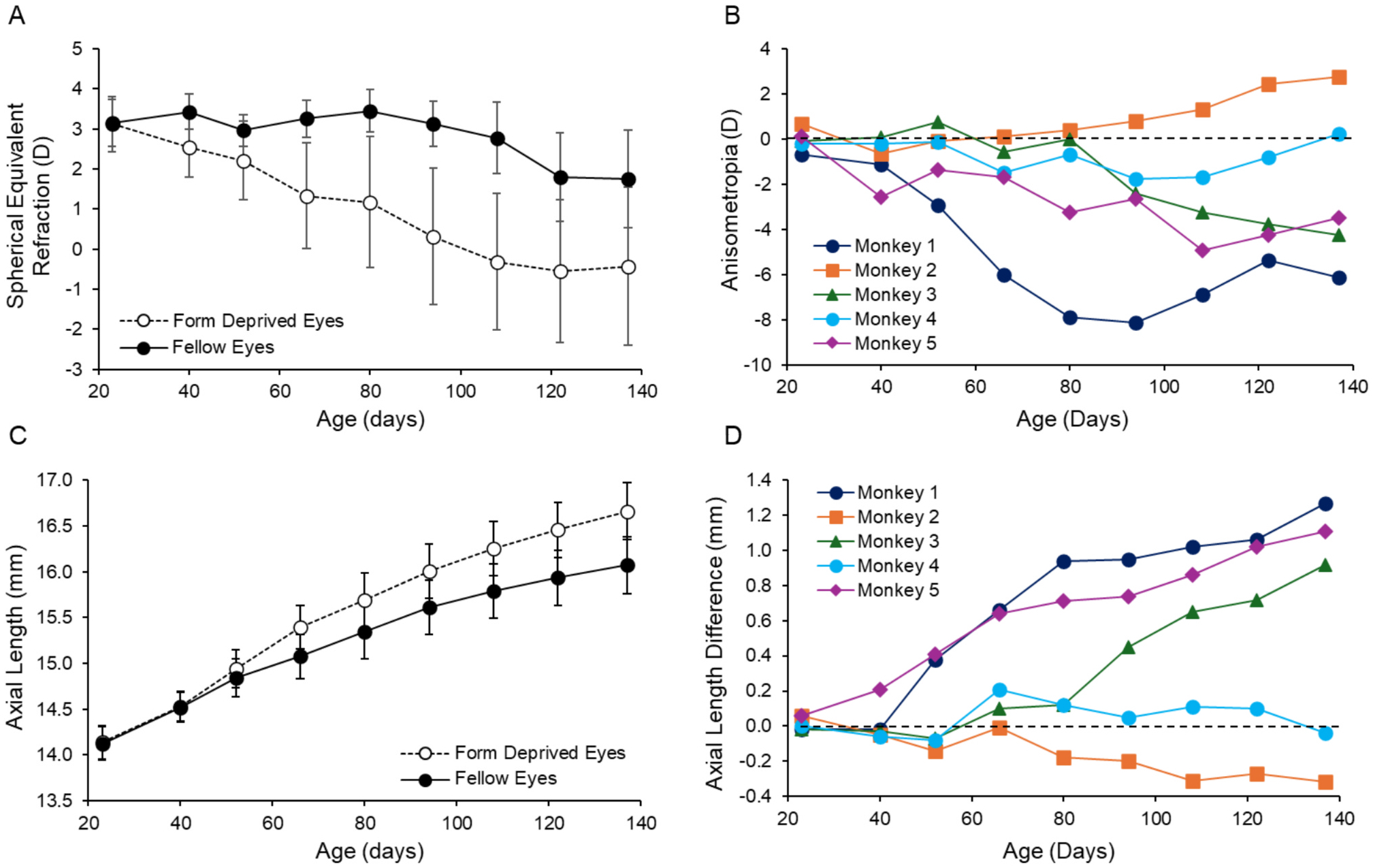
A) Mean (± standard error of the mean) spherical equivalent refraction (N = 5) and B) anisometropia for individual monkeys; C) mean (± standard error of the mean) axial length (N = 5), and D) difference in axial length between form-deprived eyes and fellow eyes for individual monkeys; dashed lines represent zero difference.

Data for individual monkeys are presented in Table 1. Mean refraction and anisometropia across the treatment period for five monkeys reared with monocular form-deprivation are shown in Fig. 2A and B, respectively. At baseline, mean form-deprived eye and fellow eye spherical equivalent refractions were +3.13 ± 1.54 D and +3.15 ± 1.31 D, respectively, with no significant difference between eyes (P = 0.93). At the end of treatment, mean refractions were −0.43 ± 4.41 D in form-deprived eyes and +1.75 ± 2.71 D in fellow eyes (P = 0.25). Mean anisometropia at end of treatment was −2.18 ± 3.60 D (range: −6.13 to +2.75 D). Three monkeys developed monocular myopia (anisometropia −3.50 D, −4.25 D, and −6.13 D), one remained emmetropic (anisometropia +0.25 D), and one developed monocular hyperopia (anisometropia +2.75 D).

Mean axial length and differences in axial length between form-deprived and fellow eyes across the treatment period are shown in Fig. 2C and 2D, respectively. At baseline, mean form-deprived and fellow eye axial lengths were 14.14 ± 0.41 mm and 14.12 ± 0.42 mm, respectively, with no significant difference between eyes (P = 0.43). At the end of treatment, mean axial lengths were 16.66 ± 0.70 mm in form-deprived eyes and 16.07 ± 0.61 mm in fellow eyes (P = 0.14). Mean axial length difference between form-deprived and fellow eyes was 0.72 ± 0.32 mm.

**Fig. 3.**
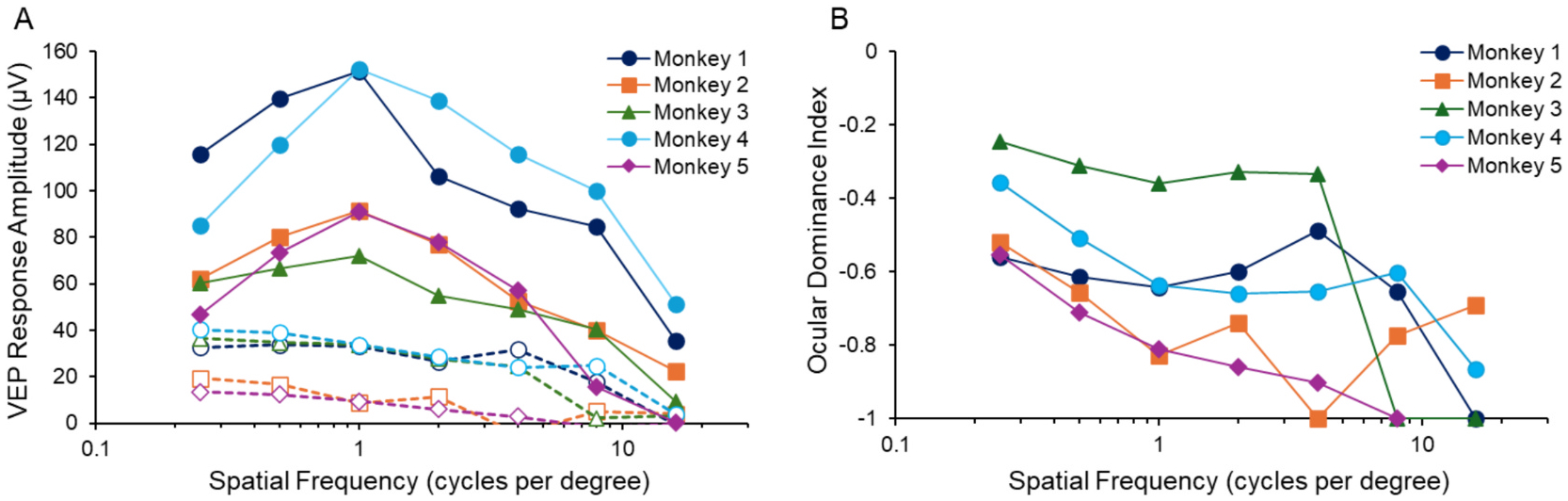
A) Mean VEP response amplitude above noise-floor for form-deprived eyes (dashed lines) and fellow eyes (solid lines), and B) ocular dominance index for individual monkeys.

Fig. 3A shows the mean VEP response above noise-floor at the different spatial frequencies from individual monkeys during monocular viewing of the form-deprived and fellow eyes. The visual cortical responsivity was significantly diminished when the form-deprived eye was stimulated versus when the fellow eye was stimulated. The preservation of measurable VEP responses at higher spatial frequencies in fellow eyes, coupled with attenuation in amblyopic eyes, is consistent with a neural (amblyopic) deficit rather than limitations imposed by optical blur alone.

**Fig. 4.**
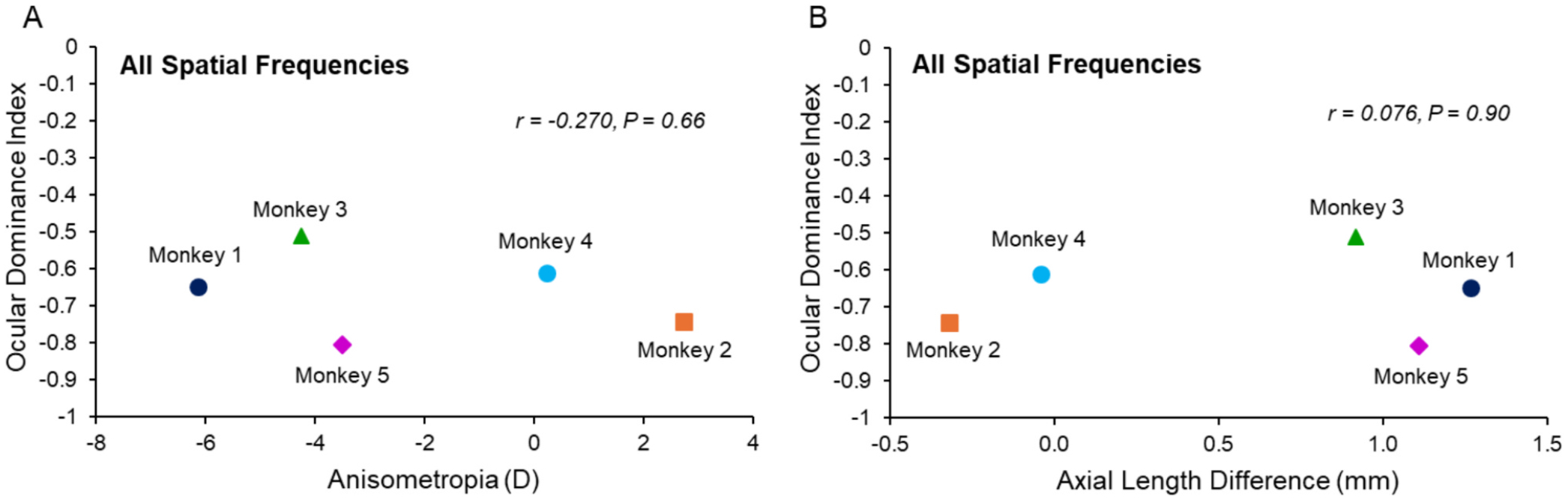
Mean ocular dominance index across all spatial frequencies with A) anisometropia and B) axial length difference between form-deprived and fellow eyes; Pearson r and P values are shown.

**Fig. 5.**
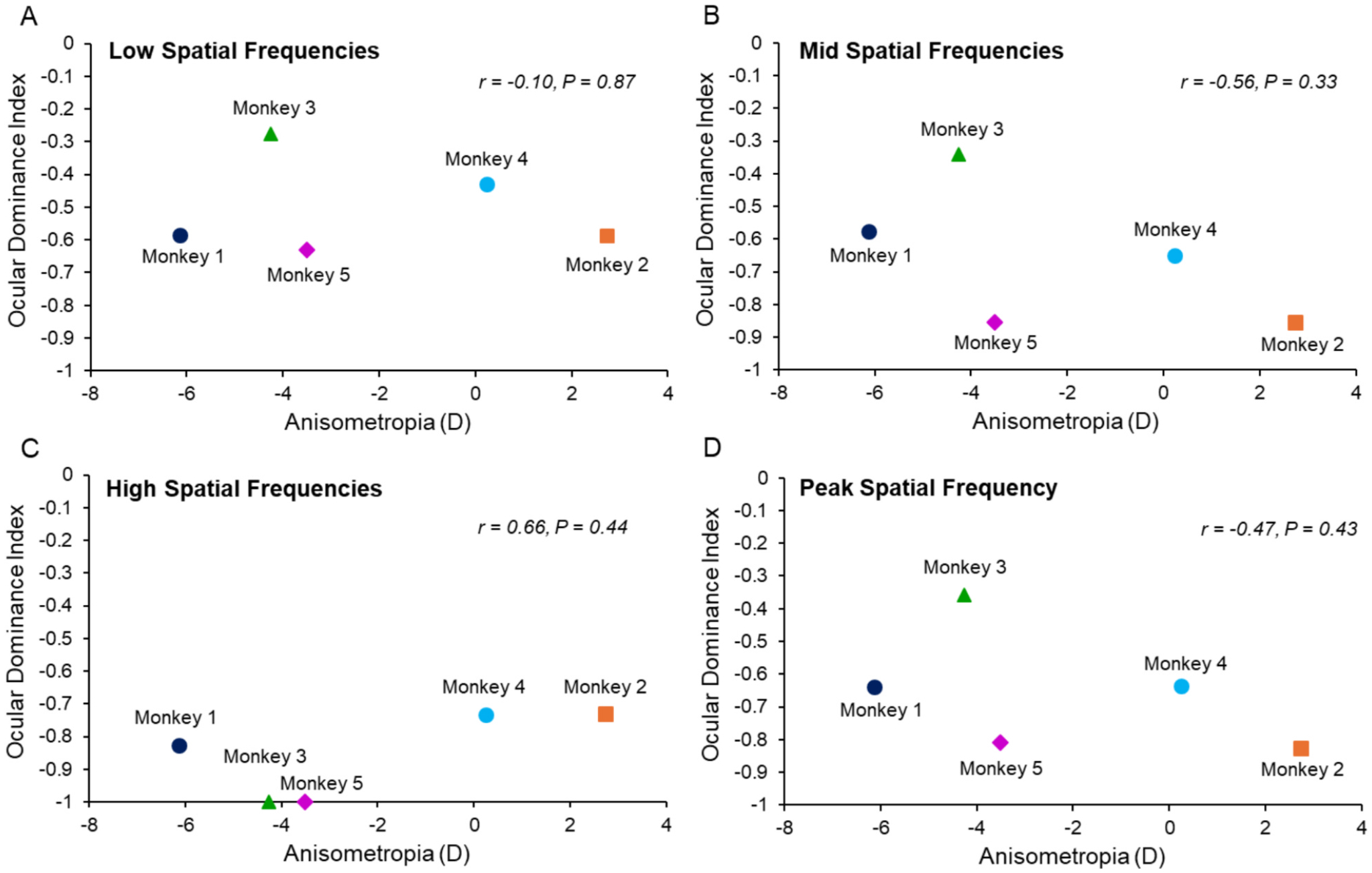
Mean ocular dominance index for A) low spatial frequencies (0.25–0.5 cyc/deg), B) mid spatial frequencies (1–4 cyc/deg), C) high spatial frequencies (8–16 cyc/deg), and D) peak spatial frequency (1 cyc/deg) with anisometropia; Pearson r and P are shown.

VEP responses were used to calculate an ODI at each testing spatial frequency (Fig. 3B). All monkeys demonstrated a significant amblyopic deficit, with a mean ODI across all spatial frequencies of −0.66 ± 0.11 (range: −0.43 to −0.81). Fig. 4 shows the relationship between cortical deficit due to amblyopia and the ocular deficit due to anisometropia and axial length. There was no correlation between the amplitude of the anisometropia and mean ODI (r = −0.270, P = 0.66) or between axial length difference (between form-deprived and fellow eyes) and mean ODI (r = 0.076, P = 0.90). Furthermore, the relationships between mean ODI for low (0.25–0.5 cyc/deg), mid (1–4 cyc/deg), and high spatial frequencies (8–16 cyc/deg), as well as for the spatial frequency at which the VEP response peaked (1 cyc/deg), with anisometropia were explored (Fig. 5). Consistent with findings from the overall mean ODI, there were no significant correlations (P > 0.05 for all).

## 4. Discussion

Monocular form-deprivation in young rhesus monkeys produced a robust amblyopic deficit in all animals as measured by pattern VEPs, despite substantial variability in refractive outcomes, ranging from myopia to hyperopia. Extending prior work that has largely examined ocular growth and visual function separately, the present study directly links refractive and biometric outcomes with electrophysiological measures of amblyopia, demonstrating that cortical deficits can occur across a wide range of refractive responses. In this cohort, the depth of the amblyopic deficit did not correlate with the magnitude of induced anisometropia. These findings suggest that pronounced deficits in cortical visual function can arise across a range of eye growth responses. However, given the limited sample size, the data do not exclude a moderate association that could not be detected. Overall, these results are consistent with the possibility that form-deprivation engages partially distinct mechanisms governing cortical function and axial growth.

Although form-deprivation has been extensively studied in non-human primates, investigations of amblyopia have relied primarily on behavioral and psychophysical assessments, mostly in adult animals, as well as single cell electrode recordings of neuronal response properties in the visual cortex (Bi, Zhang, Tao, Harwerth, Smith & Chino, 2011, Harwerth, Smith, Boltz, Crawford & von Noorden, 1983, Kiorpes, 2019, Smith, Chino, Ni, Cheng, Crawford & Harwerth, 1997). Refractive outcomes have not been directly addressed in studies examining amblyopia. The present use of VEPs to directly quantify spatial frequency-specific visual deficits represents a novel finding that demonstrates the dissociation between neural amblyopia from refractive and biometric outcomes.

Early observations by Hubel and Wiesel established that monocular deprivation during sensitive periods drives profound cortical changes in kittens (Hubel & Wiesel, 1970). Subsequent studies in non-human primates extended these observations to show that early deprivation paradigms via lid fusion produced amblyopia-like deficits (Wiesel & Raviola, 1977). Interestingly, the authors observed that with lid fusion, the eyes also significantly enlarged, thereby uncovering the paradigm of form-deprivation myopia. The present results indicate that the presence and severity of cortical deficit are not predicted by the refractive endpoint under form-deprivation. One plausible explanation is that the cortical effects of form-deprivation saturate early, whereas refractive development reflects a slower and more variable integration of retinal growth signals over time. Supporting this temporal dissociation, a guinea pig model demonstrated that amblyopia can be established within approximately one week while myopia develops more gradually over subsequent weeks (Tian, Guo, Ying, Liu, Li & Wang, 2021), implying that functional deficits precede, and do not require, significant ocular elongation. Similarly, amblyopia can be induced in non-human primates with just 2 weeks of form-deprivation when the deprivation is begun early in the critical period (Harwerth et al., 1981).

While form-deprivation myopia is now established in animal models, several studies have also demonstrated the phenomenon of form-deprivation myopia in humans. O’Leary and Millodot reported that individuals with unilateral ptosis tended to exhibit greater myopia in the occluded eye compared with the fellow eye (O’Leary & Millodot, 1979). Similarly, Hoyt et al. identified axial myopia in a subset of patients with complete congenital eyelid closure (Hoyt, Stone, Fromer & Billson, 1981). A retrospective study found that patients with unilateral ptosis showed a higher frequency of myopia in eyes with ptosis compared to the fellow eye; a subset also demonstrated amblyopia (Huo et al., 2012). In contrast, Gusek-Schneider and Martus reported that most eyes with unilateral ptosis were hyperopic, with only 15% exhibiting myopia, although myopia occurred more frequently in ptotic eyes than in fellow eyes (15% vs 4.4%) (Gusek-Schneider & Martus, 2001). Together, previous findings, along with the present results, indicate that substantial amblyopia can develop even in the absence of marked myopic shifts, supporting early detection and treatment of visual deprivation regardless of refractive status at presentation.

Visual evoked potentials are a clinical method of examining the visual pathway from the retina to the primary visual cortex, with the International Society for Clinical Electrophysiology of Vision (ISCEV) providing standard guidance on the technique (Sustar Habjan, Bach, van Genderen, Li, Mizota, Nilsson, Thompson & Robson, 2025). VEPs provide an objective, noninvasive means of assessing spatial vision beyond the limits of reliable behavioral testing, for example, in infants. The VEP is most commonly used in studies of human visual processing (Norcia, 2013), although it has also been employed for animals, including the cat (Fong et al., 2021), mouse (Gavornik & Bear, 2025), and non-human primate (Gabelt, Rasmussen, Tektas, Kim, Peterson, Nork, Ver Hoeve, Lutjen-Drecoll & Kaufman, 2012, Ghilardi, Marx, Onofrj, Glover & Bodis-Wollner, 1987, Schroeder, Tenke & Givre, 1992, Tychsen et al., 2004, Voyles & Kiorpes, 2016). In particular, pattern-reversal VEPs primarily reflect foveal and near-foveal input and depend on precise spatial phase alignment, making them especially sensitive to deficits in high spatial frequency processing. The attenuation of responses at higher spatial frequencies observed here is therefore consistent with a central amblyopic deficit, rather than a consequence of optical blur or axial elongation alone. Importantly, these reductions were evident even in animals that did not develop substantial myopia, indicating that early form-deprivation disrupts cortical spatial processing independently of eye growth. This dissociation supports the view that form-deprivation exerts parallel but partially independent effects on ocular and neural development, with pattern VEPs providing a sensitive measure of deprivation-induced deficits not captured by refractive or biometric outcomes.

In the current study, monocular form-deprivation did not reliably induce myopia in all monkeys. Of five monkeys, only three developed significant anisometropic myopia, whereas one monkey showed no response and one monkey developed anisometropic hyperopia. One monkey experienced a brief interruption in deprivation; although this monkey did not develop myopia, it demonstrated a robust amblyopic deficit, consistent with the overall dissociation between cortical and refractive outcomes. This is in accordance with the known variability of form-deprivation myopia across animal models, and particularly the rhesus monkey. In primates, form-deprivation myopia is recognized as a graded phenomenon, with the magnitude, and even direction, of refractive change varying across individuals (Smith & Hung, 2000). For example, in a study of 30 form-deprived rhesus monkeys, only 18 developed anisometropia of greater than 1 D (Qiao-Grider, Hung, Kee, Ramamirtham & Smith, 2004). Individual differences in susceptibility to image degradation are well documented in rhesus monkeys, with some animals exhibiting weak or even opposite growth responses under comparable deprivation conditions, reflecting variability in how retinal signaling pathways integrate degraded visual input with competing cues that support emmetropization. This variability reflects biologically meaningful differences in susceptibility and in how growth-control pathways weigh degraded-image cues against other visual inputs.

In a retrospective analysis, Smith, et al. investigated the relationships between emmetropization, anisometropia, amblyopia, and strabismus using data from non-human primates subjected to a range of early visual manipulations, including form-deprivation, optically imposed anisometropia, and experimentally induced strabismus (Smith, Hung, Arumugam, Wensveen, Chino & Harwerth, 2017). Their analysis demonstrated that amblyopia does not disrupt emmetropization and does not determine the direction or magnitude of ocular growth; amblyopic eyes retained the ability to detect defocus and undergo compensatory growth. Hyperopic anisometropia emerged as a primary risk factor for amblyopia, whereas strabismus, rather than amblyopia itself, was identified as a key factor capable of disrupting emmetropization and promoting anisometropia. Notably, the authors emphasized the substantial variability of refractive outcomes following form-deprivation in primates and showed that functional amblyopia can occur across a broad range of refractive states. These conclusions closely align with the present findings, in which a robust VEP-defined amblyopic deficit was observed despite highly variable refractive outcomes, with no association between the magnitude of anisometropia and amblyopic severity.

In the current study, VEPs were assessed only at the end of the deprivation period; therefore, findings do not directly address the temporal sequence of neural and refractive changes. The current results demonstrate that robust VEP-defined amblyopia can be present across a wide range of refractive outcomes, but do not establish whether cortical deficits emerge earlier or reach saturation before changes in eye growth. We speculate that neural effects emerge earlier and more rapidly than refractive changes, informed by prior longitudinal studies in our lab; experiments have shown that monkeys exposed to only four weeks of form-deprivation with a light-perception only Bangerter diffuser exhibit clear VEP-defined amblyopia when tested shortly after deprivation, indicating that functional deficits can arise early in development (Kwaw et al., 2026; ARVO e-abstract 2502).

VEPs were recorded under ketamine/xylazine sedation, which could influence neural responsiveness. However, design features minimized this possibility and reduced the likelihood of differential effects between eyes. At the doses used, sedation was stable for approximately one hour, encompassing the full VEP recording period, and physiological state was continuously monitored via heart rate. Both eyes were tested within the same session to control for between-session variability, and stimulation was alternated between eyes (right, left, right, left) to mitigate potential drift in anesthetic depth. While subtle global effects of anesthesia cannot be excluded, they would be expected to affect both eyes similarly and are therefore unlikely to account for the consistent interocular differences observed.

Some limitations should be considered. The sample size (N = 5) is typical for primate developmental studies but limits power to detect subtle structure–function relationships; nonetheless, the absence of a trend between anisometropia and the cortical deficit across a wide refractive range argues against a strong coupling between these outcomes. Additionally, the ocular dominance index derived from VEPs is an objective measure but may compress individual differences once responses are markedly reduced at high spatial frequencies. Future studies could assess the time course of VEP changes relative to refractive development, incorporate complementary behavioral measures of visual function, and compare deprivation paradigms that more selectively manipulate blur versus contrast to determine which image features most strongly drive cortical versus ocular endpoints. Finally, a no-helmet control group was not included; therefore, we cannot fully exclude subtle effects of the helmet/lens apparatus (e.g., altered field of view or reflections) on ocular or cortical development, although prior work suggests minimal independent influence of the apparatus itself.

In conclusion, early monocular form-deprivation in rhesus monkeys produced a robust amblyopic deficit that did not scale with the magnitude of induced refractive error. The dissociation between eye growth and cortical deficit supports the hypothesis that form-deprivation engages at least two partially independent pathways: one, a retinally-driven ocular growth response that is sensitive to individual susceptibility and visual context, and two, a deprivation-driven cortical reweighting (amblyopia) that may be more uniformly induced during critical periods and potentially reaches a ceiling effect over the deprivation duration used here.

## Figures and Tables

**Table 1:** Spherical equivalent refractions, anismetropia, axial length, axial length difference between eyes, and mean ocular dominance index (averaged across all spatial frequencies) for form-deprived (OD) and fellow (OS) eyes of individual monkeys.

	Spherical Equivalent Refraction	Anisometropia (OD-OS)	Axial Length	Axial Length Difference (OD-OS)	Mean Ocular Dominance Index
Monkey 1	OD: −6.63 D	−6.13 D	OD: 17.81 mm	1.27 mm	−0.65
	OS: −0.50 D		OS: 16.54 mm		
Monkey 2	OD: +4.00 D	+2.75 D	OD: 15.99 mm	−0.32 mm	−0.74
	OS: +1.25 D		OS: 16.31 mm		
Monkey 3	OD: +2.13 D	−4.25 D	OD: 16.26 mm	0.92 mm	−0.51
	OS: +6.38 D		OS: 15.34 mm		
Monkey 4	OD: +1.75 D	+0.25 D	OD: 16.63 mm	−0.04 mm	−0.61
	OS: +1.50 D		OS: 16.67 mm		
Monkey 5	OD: −3.38 D	−3.50 D	OD: 16.62 mm	1.11 mm	−0.81
	OS: +0.13		OS: 15.51 mm		
